# WOAENet: a whale optimization-guided ensemble deep learning with soft voting for uterine cancer diagnosis based on MRI images

**DOI:** 10.3389/frai.2025.1664201

**Published:** 2025-10-20

**Authors:** Omar F. Altal, Amer Mahmoud Sindiani, Hamad Yahia Abu Mhanna, Salem Alhatamleh, Mohammad Amin, Hanan Fawaz Akhdar, Rola Madain, Noor Alqasem, Faheem Zayed, Sitah Alanazi, Kholoud J. Sandougah

**Affiliations:** ^1^Department of Obstetrics and Gynecology, Faculty of Medicine, Jordan University of Science and Technology, Irbid, Jordan; ^2^Department of Medical Imaging, Faculty of Allied Medical Sciences, Isra University, Amman, Jordan; ^3^Computer Science Department, Faculty of Information Technology and Computer Sciences, Yarmouk University, Irbid, Jordan; ^4^Physics Department, Imam Mohammad Ibn Saud Islamic University (IMSIU), Riyadh, Saudi Arabia; ^5^Department of Biomedical Systems and Informatics Engineering, Hijjawi Faculty for Engineering Technology, Yarmouk University, Irbid, Jordan; ^6^Department of Obstetrics and Gynecology, Specialty Hospital, Irbid, Jordan; ^7^Department of Radiology, College of Medicine, Imam Mohammad Ibn Saud Islamic University (IMSIU), Riyadh, Saudi Arabia

**Keywords:** obstetrics and gynecology, uterine cancer, soft voting, deep learning, diagnoses, MRI

## Abstract

**Objectives:**

Uterine cancer originates from the cells lining the uterus and can develop through abnormal cell growth, potentially leading to damage in surrounding tissues and the formation of precancerous cells. Early detection significantly improves prognosis. Despite advancements in deep learning-based diagnostic methods, challenges remain, including the dependence on expert input and the need for more accurate classification models. This study aims to address these limitations by proposing a novel and efficient methodology for diagnosing uterine cancer using an integrated deep learning pipeline optimized through a nature-inspired algorithm.

**Methods:**

This study introduces the Whale Optimization Algorithm-based Ensemble Network (WOAENet), a deep learning pipeline that classifies uterine MRI into three classes: malignant, benign, and normal. The Whale Optimization Algorithm (WOA) is used to fine-tune the hyperparameters of three deep learning models: MobileNetV2, DenseNet121, and a lightweight vision model (LVM). Each model is trained with its optimized settings, and its outputs are combined using a Soft Voting Ensemble method that calculates the average of the predicted probabilities to arrive at the final classification.

**Results:**

The WOAENet framework was evaluated using a uterine cancer MRI dataset obtained from King Abdullah University Hospital. Our proposed model outperformed standard pre-trained models across several performance metrics. It achieved an accuracy of 88.57%, a specificity of 94.29%, and an F1 score of 88.54%, indicating superior performance in diagnosing uterine cancer.

**Conclusion:**

WOAENet demonstrates a high level of accuracy and reliability in classifying uterine MRI images, marking a significant advancement by utilizing a novel dataset. The findings support the potential of AI-driven approaches in enhancing the diagnosis and treatment of gynecological conditions, paving the way for more accessible and accurate clinical tools.

## 1 Introduction

One of the most common tumors of the female reproductive system is uterine cancer. It is brought on by the uterine lining’s abnormal cell proliferation, which damages the surrounding tissue as the cells divide (Esmaeilzadeh and Nasirzadeh, 2023). While uterine cancer is less common in Africa and Asia, it is more common in the Americas and Europe. Experts attribute this to environmental risk factors and obesity. Women between the ages of 40 and 60 are more likely to have the illness (Felix and Brinton, 2018). It is the fifteenth most common type of cancer in the general population (Hamoud et al., 2023). The most studied symptom of uterine cancer is abnormal uterine bleeding in premenopausal, postmenopausal, and perimenopausal women (Boeckstaens et al., 2020). Obesity is a significant risk factor for uterine cancer, with women who are overweight or obese being two to four times more likely to develop endometrial cancer compared to women with a lower body mass index (Somasegar et al., 2023).

The advancement of uterine cancer may make diagnosis and treatment more difficult, which could result in a poor prognosis. Tumor staging is therefore essential. There are three stages of this disease: low, intermediate, and high risk. Like other cancers, uterine cancer must be detected early (Dong et al., 2020). Based on their morphological and functional characteristics, analysis that depends on diagnostic accuracy can help classify tissues as either malignant or non-malignant (Keall et al., 2022). In modern clinical practice, magnetic resonance imaging (MRI) is frequently used for several purposes, such as the clinical staging of malignant tumors and the differentiation of benign from malignant gynecological problems (Lu and Broaddus, 2020). The primary method for determining the anatomical origin of uterine cancer is magnetic resonance imaging. MRI is necessary to differentiate between endometrial and cervical sources of uterine tumors when clinical and histological tests are not feasible (Gui et al., 2022). Ultrasonography is increasingly used to evaluate tissue elasticity to diagnose and treat clinical uterine cancers and other issues (Wang et al., 2022).

In recent years, artificial intelligence (AI) has been increasingly applied in medicine, particularly for diagnosis (Akazawa and Hashimoto, 2021). Deep learning and image processing techniques are utilized to improve the early detection of uterine cancer and determine if it is benign, malignant, or subclassified. Eventually, this will lead to stronger treatments that save lives (Maheswari et al., 2024). Convolutional neural network (CNN)-based deep learning approaches, also known as deep CNNs (DCNN), have recently produced impressive results in picture pattern recognition (Lundervold and Lundervold, 2019). Deep learning has been applied to a variety of computer vision applications, including segmentation (Soffer et al., 2019), classification (Fujioka et al., 2020), and lesion detection (Shin et al., 2016).

The use of MRI-based AI in gynecology for uterine cancer detection has not been adequately documented. Furthermore, some studies use a limited approach to tumor diagnosis. Therefore, this study presents an effective method that combines deep learning models and uses the WOA to optimize them. By automatically selecting the optimal set of hyperparameters, including learning rate, batch size, number of units in dense layers, and dropout rate, this method improves the performance of deep learning models. The main contributions of this paper are summarized as follows: This paper introduces a new integrated deep learning pipeline called WOAENet, which leverages the WOA for uterine cancer diagnosis.

- The outputs of the optimized models are combined using a Soft Voting Ensemble strategy, which increases classification robustness and accuracy by averaging the predicted probabilities.
- WOAENet was trained and evaluated on a new uterine MRI dataset from King Abdullah University Hospital, achieving superior performance (88.57% accuracy, 94.29% specificity, and 88.54% F1 score) compared to previously trained models.
- This study pioneers the use of deep learning to classify uterine tumors using a new dataset, highlighting the potential of artificial intelligence to improve the diagnosis and treatment of gynecological diseases.

The techniques employed, a thorough explanation of the dataset, the suggested research approach, and training regimens are all covered in Section 2. The data are analyzed, and the effectiveness of the suggested model in uterine cancer diagnostic tests is assessed in Section 3. The most significant studies in the diagnosis of uterine cancer are covered in Section 4, and Section 5 ends with crucial conclusions and recommendations for further study.

## 2 Materials and methods

**Figure 1:**
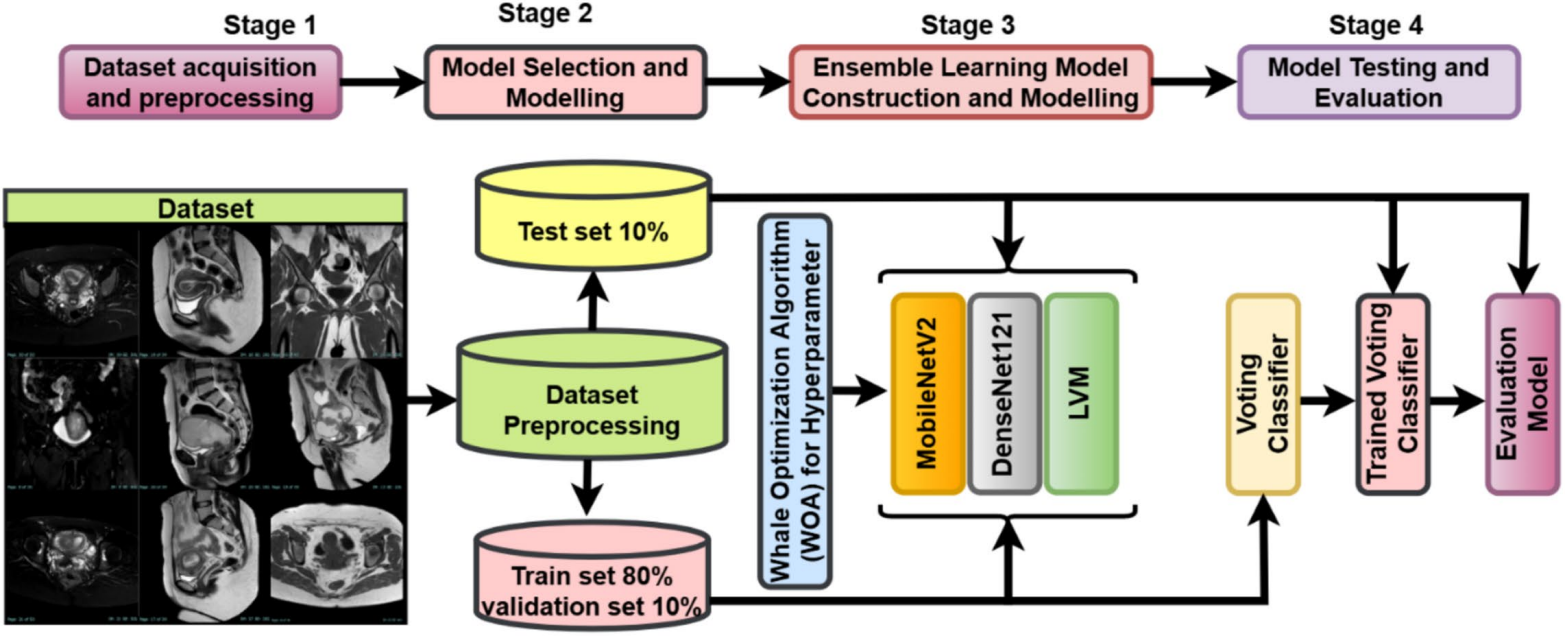
Overview of the WOAENet framework for uterine cancer MRI image classification using optimized ensemble deep learning.

The study follows a complete deep learning pipeline-based framework, WOAENet (Whale Optimization Algorithm-based Ensemble Network), optimized by the WOA algorithm for uterus image classification. As shown in Figure 1, three candidate models were built: MobileNetV2, DenseNet121, and a custom lightweight vision model (LVM). Each model contained tunable hyperparameters, such as learning rate, dropout rate, dense units, weight decay, activation function, and optimizer type. Each model contained tunable hyperparameters such as learning rate, dropout rate, dense units, weight decay, activation function, and optimizer type. All these components were controlled and fine-tuned via hyperparameters optimized using the Whale Optimization Algorithm (WOA), which was employed to minimize the validation loss by conducting an iterative population-based search over an 11-dimensional normalized hyperparameter space. The search was stopped at a small number of iterations and whales to maintain a balance between efficiency and performance. All the models were trained on a uterus MRI dataset. For performance evaluation, during WOA optimization, the candidate models were trained for only a few epochs, followed by further training of all candidates based on the very best hyperparameters found. After completing training, WOAENet applied a soft voting ensemble scheme to average class probabilities predicted by all individual models to improve robustness and classification accuracy.

### 2.1 Data acquisition

**Figure 2:**
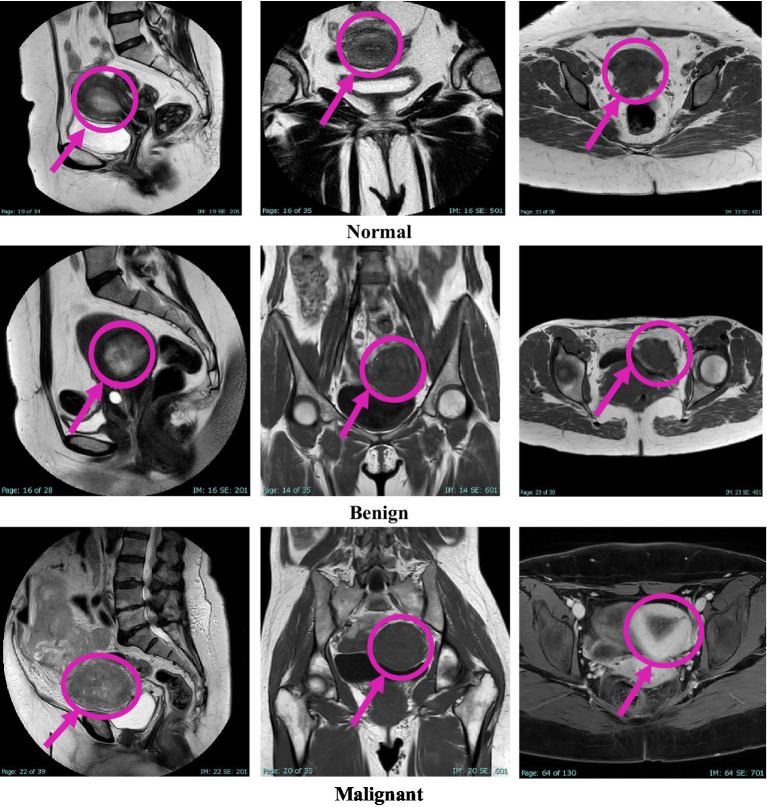
An example from the KAUH-UCM image dataset.

This study was approved by the Institutional Review Board (IRB) at King Abdullah University Hospital, Jordan University of Science and Technology (JUST). Radiologists retrospectively diagnosed patients using MRI data collected over 4 years, from early 2020 to early November 2024. Image extraction and dataset assembly were finalized during the data collection window between December 2024 and March 2025, during which anonymized and pre-evaluated images were organized into a structured dataset. The dataset comprises 1,814 MRI images collected from 450 female patients, aged between 18 and 85 years. The cases were classified into three diagnostic categories: normal, benign, and malignant, with each case represented by three imaging planes—sagittal, coronal, and axial. All images were acquired using the Ingenia Ambition 1.5 T Sand MRI scanner and exported in JPG format at a standard resolution of 720 × 720 pixels. To ensure the accuracy of the classification, KAUH obstetrics and gynecology physicians independently reviewed the imaging data. Table 1 shows the distribution of the KAUH-UCM dataset, with a representative sample from each group shown in Figure 2.

**Table 1:** The quantity and distribution of images in each KAUH-UCM category.

Case	Quantity of images
Normal	497
Benign	699
Malignant	618
Total	1,814

### 2.2 Preprocessing

In medical image analysis, and specifically in uterine cancer diagnosis via MRI, pre-processing holds paramount importance in establishing a strong foundation that will directly influence the accuracy and robustness of the classification models. This phase involves working on the input image resolution, encoding class labels, and stratified data splits to preserve class balance. To achieve class balance (699 images per class), we applied targeted data augmentation techniques—including shear, zoom, and horizontal flip—only to the training set. This was done to synthetically increase samples in underrepresented classes (normal and malignant) without duplicating existing images, avoiding potential overfitting, with 699 for each class: normal, benign, and malignant. To prevent data leakage and ensure generalizability, the data splitting was performed at the patient level. MRI images from different scanners and clinical sites are variable in size, resolution, and intensity profile (Moradmand et al., 2020). To standardize the inputs of a CNN, all images are resized to 224 × 224 fixed pixels compatible with popular pre-trained architectures such as MobileNetV2 and DenseNet121. The resizing operation is expressed in the following, as shown in Equation 1:

(1) ∀x∈X,x→Resize(x,224×224)

Beyond resizing, pixel intensity values are normalized to the range [0,1] by scaling all pixel values with a factor of $\frac{1}{255}$ , this normalization ensures numerical stability during training and helps the optimization algorithms converge more efficiently (Li et al., 2021), as shown in Equation 2:

(2) $$x_{\text{normalized}}=\frac{x_{\mathrm{raw}}}{255}$$

Overfitting reduction and increased model robustness through major data augmentation are situated within real-time training-oriented interventions considered under Keras’ Image Data Generator (Um et al., 2019). The arguments applied to the augmentations include a Shearing Transformation with shear intensity limited to 0.2 to mimic minor affine distortions, slight irregularities or imperfections in shape, Random Zooming while zooming on random areas of the image to help the model identify localized tumor features at varying scales, Horizontal Flipping: allowing random horizontal flips, whereby the model learns spatially invariant features, i.e., recognizing patterns that are symmetric to one another. Essentially, these augmentations generate great diversity and variability for the training data and enhance the generalization of the models to unseen cases. With the three categories for each MRI scan class being clinically relevant, normal, benign tumor, or malignant tumor, the categorical labels are transformed into integer indices for the model, as shown in Equation 3:

(3) $$\text{Label}\_\text{Index}(y)=\{\begin{array}{ll} 0, & \text{if}\;y=\text{Normal}\\ 1, & \text{if}\;y=\text{Benign}\\ 2, & \text{if}\;y=\text{Malignant} \end{array}$$

Such encoding permits the network to consider labels as numerical tensors during training and evaluation. To avoid systematic biases and guarantee balance of representations across classes, the entire dataset is divided into train, validation, and test subsets while keeping the same class distributions (stratified splitting). The training set, $D_{\text{train}}$ , is formed from 80% of the full data. The validation set, $D_{\mathrm{val}}$ comprises 10%. Test set, $D_{\text{test}}$ comprises 10%. If $N_{k}$ is the number of samples in class k , the split obeys the, as shown in Equation 4, 5:

(4) $$D={\left\{(x_{i},y_{i})\right\}}_{i=1}^{N}$$

(5) $$|D_{\text{train}}^{k}|=0.8N_{k},\;\;|D_{\mathrm{val}}^{k}|=0.1N_{k},\;\;|D_{\text{test}}^{k}|=0.1N_{k}$$

Each subset is therefore a true representation of the entire dataset, ensuring no bias is created for the majority classes and thus trustworthy evaluation metrics. Preprocessing for uterine tumor MRI images consists of uniform resizing, pixel normalization, advanced data augmentation, error-free label encoding, and balanced data splitting. Together, these steps enrich model stability, improve generalization, and build a strong footing toward downstream classification tasks.

### 2.3 Whale optimization algorithm (WOA) for hyperparameter tuning

The Whale Optimization Algorithm (WOA) is used as a nature-inspired metaheuristic optimization tool within this study for the automated hyperparameter tuning of deep networks classifying uterine tumors from MRI images (Mirjalili and Lewis, 2016). Accurate classification depends not only on very high-capacity models, but also, more importantly, on the hyperparameter choices such as learning rates, batch sizes, regularization strengths, dropout rates, and architectural parameters such as the number of convolutional filters or dense units (Brodzicki et al., 2021). These hyperparameters greatly influence the model’s generalization ability, especially when competing with complicated, high-dimensional medical imaging data, such as MRI scans.

Traditional tools such as grid-search methods or manual examinations have become almost impossible in this modern context, simply due to prohibitive computational costs plaguing them (Brodzicki et al., 2021). WOA, thus, solves the problem by conjecturing therein an intelligent exploration of the high-dimensional parameter space, with the behavior of humpback whales searching for food in the natural world being the inspiration.

#### 2.3.1 Motivation for metaheuristic-based optimization

In MRI tumor classification, a series of challenges are faced: high variability in anatomical structures, a limited dataset, and a need to generalize models strongly. In the application concerned, the hyperparameter shows a non-linear correlation with performance, interdependence, which renders brute force methods practically helpless (Nadimi-Shahraki et al., 2021). Thus, metaheuristic algorithms like the WOA are fitted with respect to ensure the escape from local minima and to perform efficient global search without the need to keep track of gradient information or convex assumptions.

#### 2.3.2 Mathematical modeling of WOA

The Whale Optimization Algorithm (WOA) simulates the bubble-net hunting strategy of humpback whales and involves three primary mechanisms: to encircle the prey (Rana et al., 2020), to bubble-net attack (exploit), and to search for prey (explore). Here, the solution space is defined by $R^{d}$ , where d is the number of hyperparameters needing optimization. Each whale in the population stands for a possible solution vector $\vec{X}\in R^{d}$ .

Encircling Prey (Exploitation), the whales regard the current best solution as the prey and update their positions accordingly (Nadimi-Shahraki et al., 2023). Where ${\vec{X}}^{*}$ is the position of the best solution obtained so far. $\vec{X}(t)$ is the current location of the whale. $\vec{A}=2a\cdot {\vec{r}}_{1}-a$ , $\vec{C}=2\cdot {\vec{r}}_{2}$ are coefficient vectors, a is a linearly decreased factor from 2 to 0 throughout iterations, and ${\vec{r}}_{1},{\vec{r}}_{2}∼U(0,1)$ are random vectors, as shown in Equations 6, 7. The mechanism traverses toward intensification (local search) as the whales try to move toward the best solution.

(6) $$\vec{D}=|\vec{C}\cdot {\vec{X}}^{*}-\vec{X}(t)|$$

(7) $$\vec{X}(t+1)={\vec{X}}^{*}-\vec{A}\cdot \vec{D}$$

Bubble-Net Attacking Strategy (Exploitation) this simulates the spiral-shaped bubble-net behavior. Where b is a constant defining the logarithmic spiral shape (commonly set to 1), l∼U[−1,1] is a random number. The algorithm stochastically chooses between spiral update and encircling with probability p∈[0,1] , as shown in Equation 8. This probabilistic behavior enhances the search diversity and mimics the natural behavior of whales, varying between exploration and exploitation.

(8) $$\vec{X}(t+1)=\vec{D}\cdot e^{\mathrm{bl}}\cdot \cos(2\mathrm{\pi l})+{\vec{X}}^{*}$$

Searching for Prey (Exploration) if $|\vec{A}|\geq 1$ , the whale randomly chooses another whale and updates its position. Here, ${\vec{X}}_{\mathrm{rand}}\;$ is a randomly selected whale (Abualigah et al., 2024). This mechanism ensures the exploration of the global search space to avoid premature convergence, as shown in Equations 9, 10:

(9) $$\vec{D}=|\vec{C}\cdot {\vec{X}}_{\mathrm{rand}}-\vec{X}(t)|$$

(10) $$\vec{X}(t+1)={\vec{X}}_{\mathrm{rand}}-\vec{A}\cdot \vec{D}$$

#### 2.3.3 Fitness function for hyperparameter optimization

The fitness of each whale (candidate hyperparameter set) is evaluated using a partial training strategy (Pham et al., 2020), where a deep learning model (MobileNetV2, DenseNet121, or LVM) is trained for a limited number of epochs 10 epochs, and the validation loss is recorded as the objective function (Mohammed et al., 2019). Where $\vec{X}\in R^{11}$ is the hyperparameter vector, $L_{\mathrm{val}}$ is the validation loss, θ are the model parameters, as shown in Equation 11. This formulation allows WOA to identify hyperparameter configurations that minimize validation loss and hence maximize generalization on unseen MRI scans.

(11) $$\text{Fitness}(\vec{X})=L_{\mathrm{val}}(\theta ;\vec{X})$$

#### 2.3.4 Parameter encoding and normalization

Each dimension in the whale’s position vector $\vec{X}$ corresponds to a hyperparameter. To ensure scalability and uniformity in the search, all hyperparameters are normalized to [0,1] and decoded during evaluation. For example Nadimi-Shahraki et al. (2023), such normalization allows WOA to operate uniformly across parameters with different physical scales and types, as shown in Equations 12–14:

(12) $$\mathrm{Learning rate:}\mathrm{lr}=10^{-5+3x_{0}}$$

(13) $$\mathrm{Dropout:}\mathrm{dr}=0.1+0.4x_{2}$$

(14) $$\mathrm{Dense units:}\mathrm{du}=64+448x_{3}$$

To appropriately tune the deep learning architectures for uterine tumor classification from MRI images, the WOA was used to perform a search on a multidimensional hyperparameter space. Table 2 summarizes the hyperparameters that were optimized with WOA, along with their corresponding search ranges and encoding strategies. This set includes learning rate, batch size, dropout rate, and number of dense units, along with convolutional filters (specific to the LVM model), as well as categorical variables such as optimizer and activation function (Aljarah et al., 2018). The normalized search spaces were mapped onto [0,1] and decoded accordingly during every fitness evaluation to allow thorough investigation of the configuration landscape.

**Table 2:** Hyperparameter search space for WOA-based deep learning optimization.

Hyperparameter	Search range/values	Encoding/notes
Learning rate	$10^{-5}$ – $10^{-2}$	$\mathrm{lr}=10^{-5+3x}$ , log-scaled
Batch size	16–128	bs=int(16+112x) , linear
Dropout rate	0.1–0.5	dr=0.1+0.4x , linear
Dense units	64–512 (integers)	du=int(64+448x) , linear
Optimizer type	{Adam, SGD, RMSprop}	*Index:* idx=round(2x)∈{0,1,2}
Activation function	{ReLU, LeakyReLU, ELU}	*Index:* idx=round(2x)∈{0,1,2}
Weight decay (L2)	$10^{-6}$ – $10^{-3}$	$\mathrm{wd}=10^{-6+3x}$ *, log-scaled*
Momentum (SGD only)	0.5–0.95	*Only used if optimizer = SGD:* mom=0.5+0.45x
Conv1 filters (LVM)	16–96	$f_{1}=\mathrm{int}(32\times (0.5+1.5x))$
Conv2 filters (LVM)	32–192	$f_{2}=\mathrm{int}(64\times (0.5+1.5x))$
Conv3 filters (LVM)	64–384	$f_{3}=\mathrm{int}(128\times (0.5+1.5x))$

#### 2.3.5 Computational efficiency and convergence

Since deep learning algorithms have more computational requirements, the number of whales (2–5) and iterations (5–10) were selected based on preliminary trials that aimed to minimize computational overhead while maintaining classification performance. These values proved sufficient for stable convergence due to the limited feature dimensionality and the pre-trained nature of the backbone network (Nadimi-Shahraki et al., 2023). The optimization process terminated either when the maximum number of iterations was reached or when no improvement in validation loss was observed for several consecutive iterations (early stopping). WOA stays efficient due to its good exploration and exploitation balance and its applicability to non-differentiable and noisy objective spaces, which generally characterize deep learning hyperparameter landscapes. Convergence behavior is monitored through a fitness curve. Here, T represents the total number of iterations, as shown in Equation 15. Such a curve provides insights into the sequence of optimization steps taken and how stable the search process has been.

(15) $$\text{Convergence Curve}={\left[\min_{i}\text{Fitness}({\vec{X}}_{i}^{\left(t\right)})\right]}_{t=1}^{T}$$

**Figure 3:**
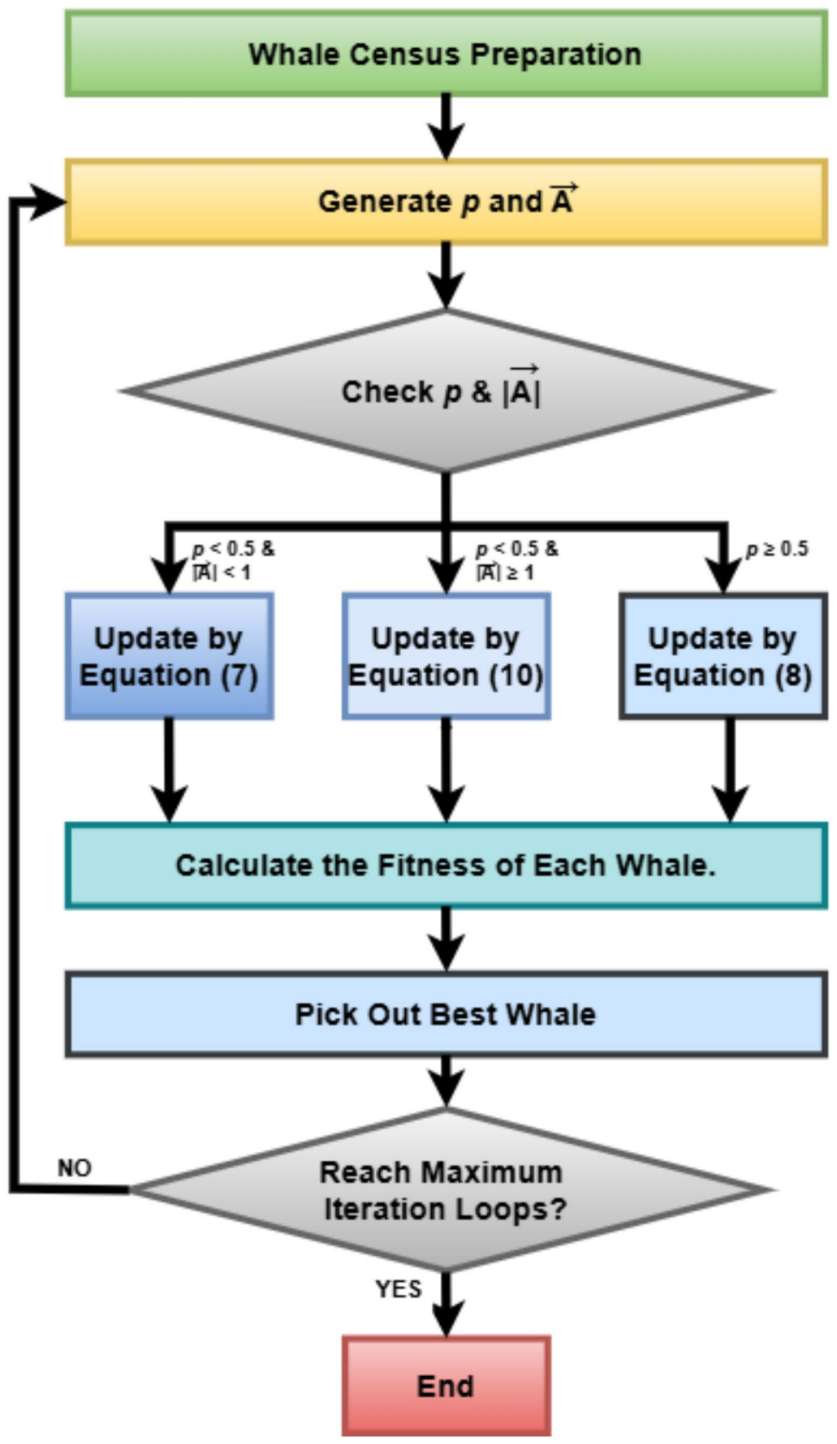
Flowchart of the whale optimization algorithm (WOA) for iterative solution refinement.

Integrating WOA into the hyperparameter tuning pipeline turned out to be a more scalable and flexible system capable of autogenerating optimized deep learning models for the classification of uterine tumors from MRI images (Liu and Zhang, 2022). By way of contrast with grid search, which is exhaustive, and manual trial-and-error methods, WOA substantially improves model performance while improving generalization and minimizing training costs. That is particularly important in medical imaging, where diagnosis may favor or disfavor a clinician depending on how brain power is expended. Figure 3 represents the basic steps of the Whale Optimization Algorithm (WOA).

### 2.4 Model architectures

Three CNN architectures, MobileNetV2, DenseNet121, and an in-house Lightweight Vision Model (LVM), were adopted to classify uterine cancer based on MRI images. Each of the architectures is parameterized and dynamically instantiated based on hyperparameters tuned with the WOA, including the learning rate, dropout rate, dense units, activation functions, and regularization strength. Hence, the design allows for flexibility, scalability, and adaptability of the model to domain-specific data such as MRI scans, which require careful treatment of spatial and structural features.

#### 2.4.1 MobileNetV2 model

MobileNetV2 is a lightweight deep convolutional neural network architecture specifically meant for efficiency on mobile and embedded platforms (Sandler et al., 2018), while maintaining a good level of performance on image classification problems. Considering uterine tumor classification with MRI images, MobileNetV2 thus stands as a strong backbone, for it seems to be the only one that balances the computational efficiency and representational power that are crucial in medical image analysis with constrained annotated data. MobileNetV2 improves upon its predecessor by introducing two key innovations: inverted residuals with linear bottlenecks and depth-wise separable convolutions. Each block in MobileNetV2 is defined by an inverted residual structure wherein the input and output are thin bottleneck layers, and the intermediate expansion layer is of high dimensionality. Hence, features are preserved at a low computational cost.

Let $x\in R^{H\times W\times C}$ be the input tensor, where H , W , and C are the height, width, and number of channels, respectively. Each MobileNetV2 bottleneck block applies the following transformations: expansion (Pointwise Convolution) where t is the expansion factor (typically t=6 ) as Equation 16, depthwise convolution as in Equation 17, projection (Linear Pointwise Convolution) as Equation 18, and residual connection as Equation 19. This inverted residual block allows the network to maintain gradient flow, preserve spatial features, and reduce the number of parameters and operations (Dong et al., 2020).

(16) $$x_{1}=\text{ReLU}6(x*W_{1}),W_{1}\in R^{1\times 1\times C\times \mathrm{tC}}$$

(17) $$\begin{array}{l} x_{2}=\text{ReLU}6(\text{DWConv}(x_{1})),\\ \;\;\;\;\;\;\;\;\;\;\;\;\text{DWConv}:\text{separate kernel}\;\mathrm{per}\;\text{channel} \end{array}$$

(18) $$x_{3}=x_{2}*W_{3},W_{3}\in R^{1\times 1\times \mathrm{tC}\times C\prime }$$

(19) $$y=x+x_{3},\text{if stride}=1\;\text{and}\;C=C\prime$$

Model customization with WOA in this study, MobileNetV2 is used as a feature extractor by setting include_top = False and freezing the pretrained layers (weights initialized on ImageNet). The extracted features are passed through.

Global average pooling. This reduces each channel to a single value, lowering overfitting risk and model complexity, as shown in Equations 20, 21. Dense layer (WOA-optimized units u∈[64,512] ) where ϕ is an activation function (ReLU, LeakyReLU, or ELU) selected by WOA. Dropout layer (WOA-optimized rate d∈[0.1,0.5] ) to reduce overfitting (Xiang et al., 2019), as shown in Equations 22, 23. Softmax classification layer where K=3 is the number of tumor classes (Benign, Malignant, Normal).

(20) $$z=\frac{1}{H\cdot W}\sum_{i=1}^{H}\sum_{j=1}^{W}x_{i,j}$$

(21) h=ϕ(Wz+b)

(22) h′=Dropout(h,rate=d)

(23) $${\hat{y}}_{k}=\frac{e^{o_{k}}}{\sum_{j=1}^{K}e^{o_{j}}},\text{for}\;k=1,2,\ldots ,K$$

**Figure 4:**
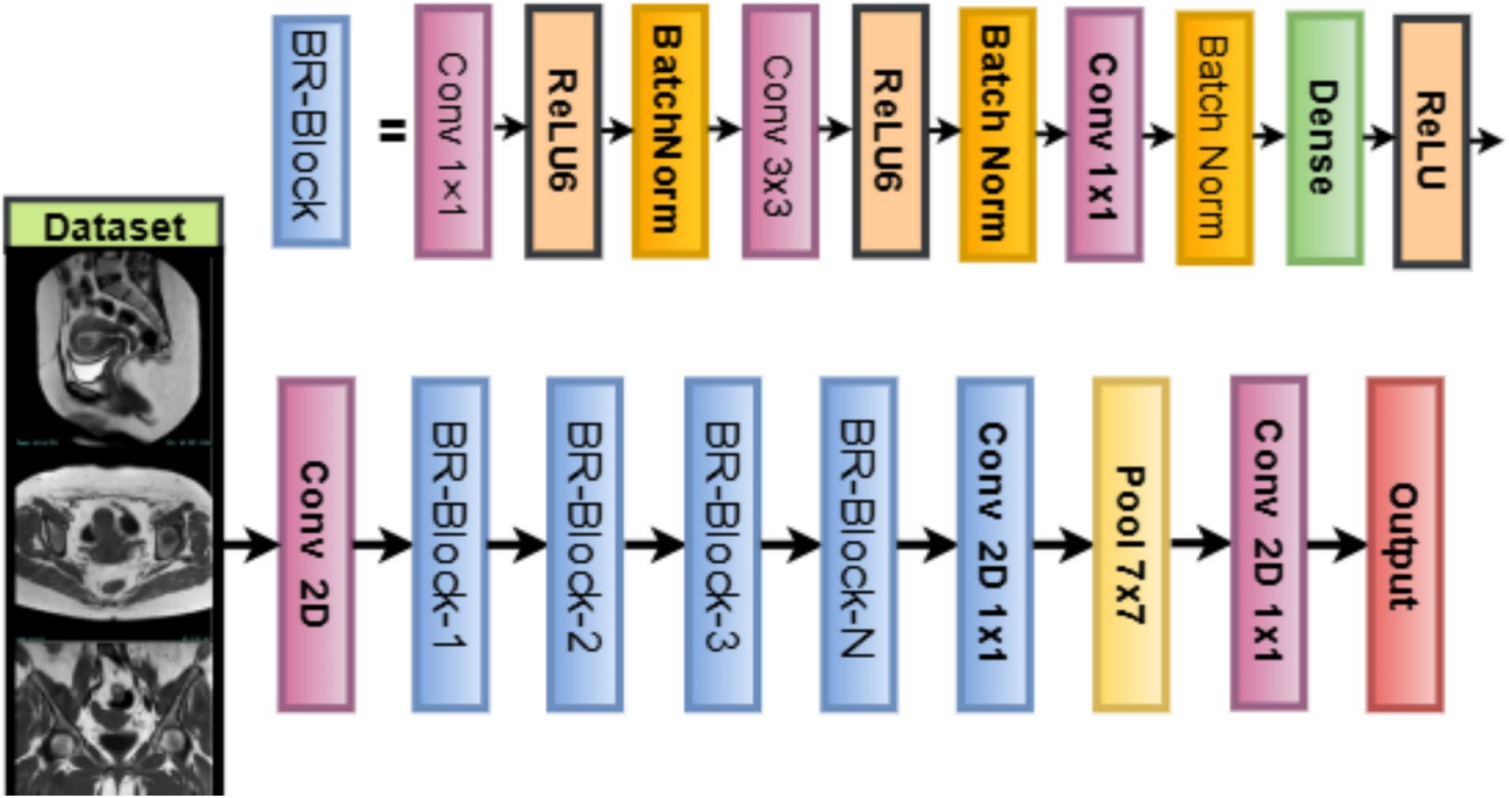
MobileNetV2 model structure.

Training configuration of the network is compiled using the Adam optimizer or alternatives (SGD, RMSprop) as determined by WOA. The loss function used is sparse categorical cross-entropy, where y is the true class index. The learning rate, batch size, regularization weight decay, and other hyperparameters are dynamically chosen by the WOA metaheuristic, ensuring model robustness and optimal convergence during training. Figure 4 illustrates the working architecture of the model, as shown in Equation 24:

(24) $$L=-\log({\hat{y}}_{y})$$

#### 2.4.2 DenseNet121 model

DenseNet121 is a Dense Connected Convolutional Network deep structural architecture designed for maximum feature reuse and rampant gradient propagation in disjoint data communities like uterine MRI (Swaminathan et al., 2021). In this work, DenseNet121 is used as the backbone network to extract high-level features that discriminate between benign, malignant, and normal uterine tumors for classification.

Contrary to other conventional CNN architectures, where each layer takes input only from its preceding layer, DenseNet connects each layer to all its preceding layers in a feed-forward fashion. This means the actual input to any layer l consists of the feature maps of all preceding layers $x_{0},x_{1},\ldots ,x_{l-1}$ from previous layers Hewlett-Packard (Liu et al., 2021). $H_{l}(\cdot )$ represents some composite function of operations of the form Batch Normalization → ReLU → Convolution. [·] indicates concatenation, rather than summation, as shown in Equation 25. By using dense connectivity, this strengthens the gradient flow through the network. Moreover, it encourages feature reuse, thereby cutting down on the total number of parameters. Thus, this also alleviates the problem of gradient vanishing, especially for very deep networks such as DenseNet121. It starts with a convolution and pooling layer. Then, four dense blocks with transition layers (1 × 1 conv + 2 × 2 average pooling) after each one. Finally, it uses global average pooling and a fully connected softmax layer. They are distributed among the four dense blocks as 6, 12, 24, and 16 layers, respectively.

(25) $$x_{l}=H_{l}([x_{0},x_{1},\ldots ,x_{l-1}])$$

For feature extraction and customization for this classification task, we utilize DenseNet121 pretrained on ImageNet as a frozen feature extractor (include_top = False). The last convolutional block output is passed through a GlobalAveragePooling2D layer, where $z_{c}$ is the pooled feature for channel c , $x_{i,j,c}$ is the activation at spatial location (i,j) in channel c , H and W are height and width of the feature map (Uemura et al., 2020). This operation reduces spatial dimensions, producing a vector of size equal to the number of channels, improving generalization and reducing overfitting. This operation reduces spatial dimensions, producing a vector of size equal to the number of channels, improving generalization and reducing overfitting, as shown in Equation 26:

(26) $$z_{c}=\frac{1}{H\cdot W}\sum_{i=1}^{H}\sum_{j=1}^{W}x_{i,j,c}$$

WOA-optimized classification heads the extracted features are passed through a classification head that is parameterized dynamically using WOA Dense Layer ϕ activation function (ReLU, LeakyReLU, or ELU), $H_{1}\in R^{u\times d}$ : weight matrix (with u∈[64,512] ), e pooled features from DenseNet backbone, as shown in Equations 27, 28. Dropout p∈[0.1,0.5] dropout rate optimized by WOA. Softmax Output Layer K=3 number of uterine tumor classes and $o_{k}$ logit corresponding to class k . Loss Function $y_{k}$ is the one-hot encoded true label (Zhang et al., 2019), as shown in Equations 29, 30. Optimizer chosen among {Adam, SGD, RMSprop} as per WOA-optimized index. Regularization L2 weight decay (search range $10^{-6}$ to $10^{-3}$ ) applied on trainable dense weights, as shown in Equation 31:

(27) $$h=\phi (H_{1}e+b_{1})$$

(28) h′=Dropout(h,rate=p)

(29) $${\hat{y}}_{k}=\frac{\exp(o_{k})}{\sum_{j=1}^{K}\exp(o_{j})}$$

(30) $$L_{\mathrm{CE}}=-\sum_{k=1}^{K}y_{k}\log({\hat{y}}_{k})$$

(31) $$L_{\text{total}}=L_{\mathrm{CE}}+\lambda ∥W_{1}{∥}_{2}^{2}$$

**Figure 5:**
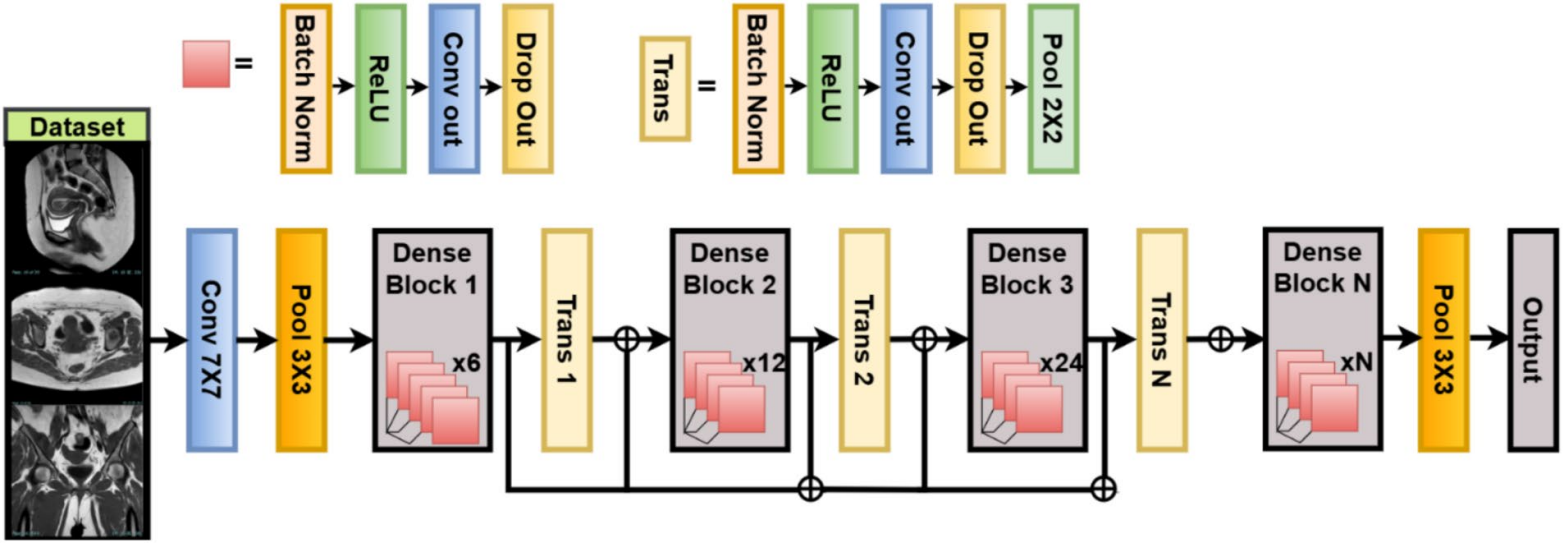
DenseNet121 model structure.

DenseNet121 has many advantages when used in the classification of uterine tumors from MRI. Increased feature propagation enables improved encoding of tissue textures and the lesion border. Fewer parameters mean better training with the given medical data since it is fewer. WOA-based parametrization helps adapt the architecture, so it generalizes best for the dataset at hand. Figure 5 illustrates the working architecture of the model.

#### 2.4.3 Lightweight vision model (LVM)

For scenarios with limited computing machines or smaller datasets that are mostly found in medical imaging facilities, this study introduces a custom-built Lightweight Vision Model (LVM) (Jiang et al., 2025). The LVM is a modular, parameterizable convolutional neural network designed for uterine tumor classification from MRI images. While it could have used large-scale pretrained models, training the LVM from scratch allows it to fine-tune itself directly on the texture and contrast patterns inherent to uterine MRI images.

The LVM architecture follows a typical hierarchical paradigm of feature extraction, having three convolution + pooling blocks arranged in series, followed by a fully connected classifier. This allows for the extraction of low-level features such as edges and textures, along with high-level features that involve shapes and boundaries important for tumor detection. Parameters in each layer, such as the number of filters, activation function, and dropout rate, are all subject to optimization based on the Whale Optimization Algorithm (WOA) so that the best validation results may be achieved (Zhang et al., 2023). This ensures a trade-off between simplicity and robustness in classification, especially when imbalanced or small datasets in the medical domain are considered.

Network architecture and equations let the input image $X\in R^{224\times 224\times 3}$ be an RGB MRI slice. The model consists of convolutional block 1, $F_{1}\in [16,96]$ : number of filters (WOA-tuned) ϕ : Activation function (ReLU / LeakyReLU /ELU), as shown in Equations 32, 33. Output shape $112\times 112\times F_{1}$ . Convolutional block 2 $F_{2}\in [32,192]$ WOA-optimized, as shown in Equations 34, 35. Convolutional block 3 $F_{3}\in [64,384]$ : WOA-optimized, output from $A^{\left(3\right)}\in R^{28\times 28\times F_{3}}$ , as shown in Equations 36, 37:

(32) $$Z^{\left(1\right)}=\phi (X*W^{\left(1\right)}+b^{\left(1\right)}),W^{\left(1\right)}\in R^{3\times 3\times 3\times F_{1}}$$

(33) $$A^{\left(1\right)}=\text{MaxPool}(Z^{\left(1\right)},2\times 2)$$

(34) $$Z^{\left(2\right)}=\phi (A^{\left(1\right)}*W^{\left(2\right)}+b^{\left(2\right)}),W^{\left(2\right)}\in R^{3\times 3\times F_{1}\times F_{2}}$$

(35) $$A^{\left(2\right)}=\text{MaxPool}(Z^{\left(2\right)},2\times 2)$$

(36) $$Z^{\left(3\right)}=\phi (A^{\left(2\right)}*W^{\left(3\right)}+b^{\left(3\right)}),W^{\left(3\right)}\in R^{3\times 3\times F_{2}\times F_{3}}$$

(37) $$A^{\left(3\right)}=\text{MaxPool}(Z^{\left(3\right)},2\times 2)$$

Following the final convolutional block of the LVM, the output feature maps are flattened into a one-dimensional vector for classification purposes (Fan et al., 2023). This flattened feature vector then passes through one fully connected dense layer whose number of output units is treated as a hyperparameter, ranging from 64 to 512. The activation function used here is selected through WOA; under different scenarios, it can be ReLU, Leaky ReLU, or ELU. The dropout layer has been included after dense transformations to avoid overfitting. The dropout rate is set in the range of 0.1–0.5. The final classification is done through a softmax layer to produce probability scores on the three classes of uterine tumors: benign, malignant, and normal. The predicted class will be the one having the highest softmax score.

**Figure 6:**
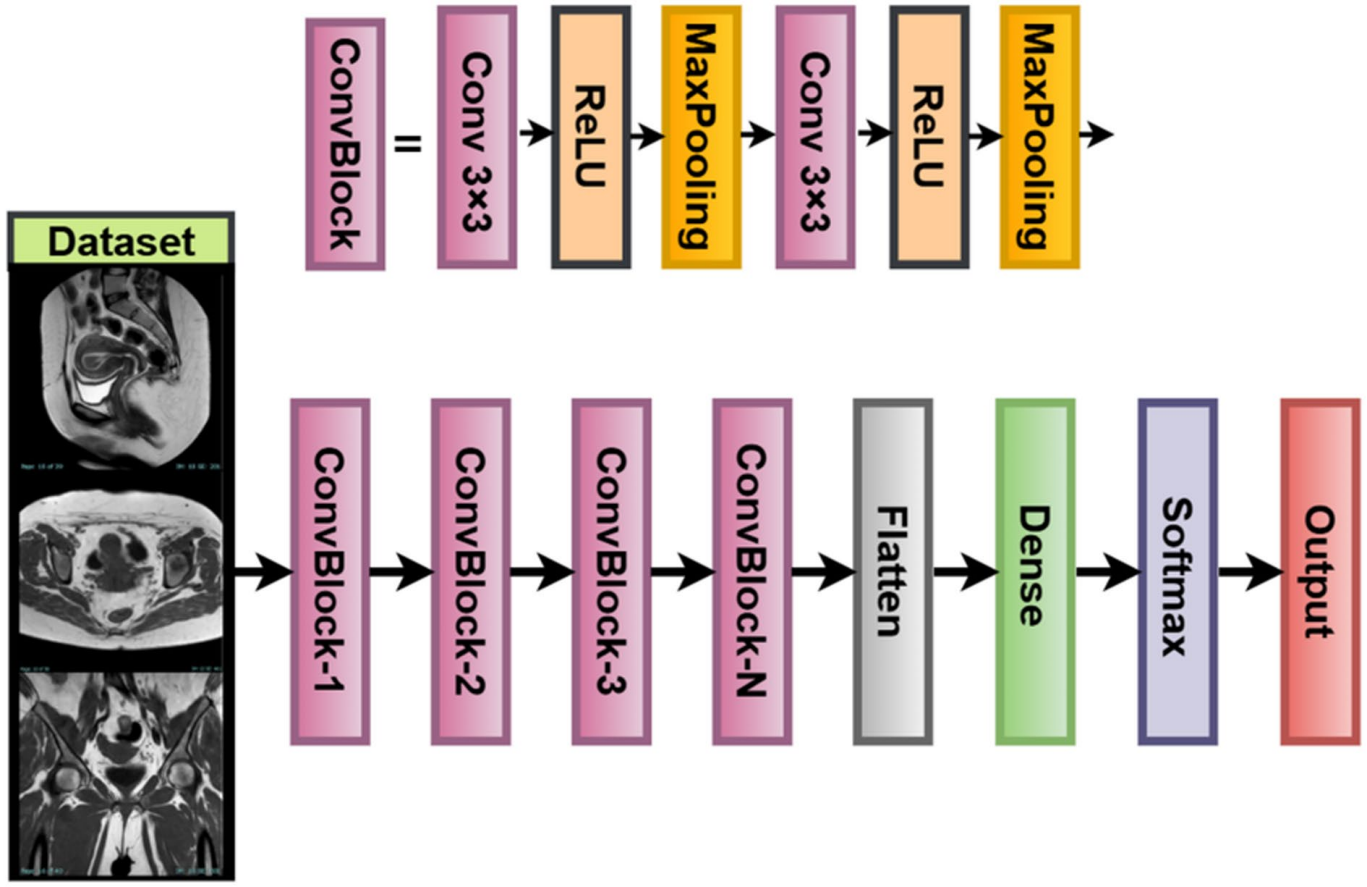
LVM model structure.

The training of the model is achieved by minimizing the sparse categorical cross-entropy loss function between the predicted probability distribution and the true class label (Nie et al., 2023). Also, L2 regularization (weight decay) is applied to every trainable layer of the network, with the coefficient *λ* being optimized by the WOA as well. Regularization prevents over-fitting by penalizing large magnitudes of weights, thereby encouraging models to behave more generally. LVM for Medical Imaging is fully customizable. Your WOA will allow you to adapt filters, activation functions, dropout rate, or dense units. The model is lightweight with a minimal memory footprint, making it suitable for developing real-time diagnostics and mobile applications in clinical environments. It also learns directly from MRI data without bias induced by pretrained natural image datasets. LVM is a flexible and interpretable alternative to deep pretrained models while facilitating domain-specific tunings for optimizing accuracy and resource usage in uterine tumor classification. Figure 6 illustrates the working architecture of the model.

### 2.5 Ensemble via soft voting

The proposed ensemble learning strategy is based on soft voting and aims to enhance the classification performance, improve stability, and increase diagnostic robustness in the automatic detection of uterine tumors from MRI images (Salur and Aydın, 2022). Ensemble models use the strength that comes from diversity among different classifiers to achieve higher generalizations and accuracies than any individual classifier. Within the framework, three diverse CNNs-MobileNetV2, DenseNet121, and a custom-built Lightweight Vision Model (LVM)-are individually trained and optimized with Whale Optimization Algorithm and then combined, through soft voting, to give the final classification output.

Each model outputs the probability distribution of classes for an input image. Let there be M models in an ensemble; then the model m is expected to produce the predicted probabilities vector $p^{\left(m\right)}=[p_{1}^{\left(m\right)},p_{2}^{\left(m\right)},\ldots ,p_{C}^{\left(m\right)}]$ with C denoting the total number of classes: in this case C=3 (Benign, Malignant, Normal), and $\sum_{k=1}^{C}p_{k}^{\left(m\right)}=1$ . The soft voting mechanism determines the average predicted probability for each class from all models, and the final predicted class $\hat{y}$ is given by the index max of the average probability (Jaradat et al., 2024). This ensures that output from each model has a say in the final decision and that class probabilities correspond to the ensemble’s head count confidence, as shown in Equations 38, 39:

(38) $${\overset{ˉ}{p}}_{k}=\frac{1}{M}\sum_{m=1}^{M}p_{k}^{\left(m\right)},\text{for}\;k=1,2,\ldots ,C$$

(39) $$\hat{y}=\arg\max_{k}{\overset{ˉ}{p}}_{k}$$

Generalization is enhanced by building an ensemble out of different architectures, thus reducing variance and model-specific overfitting. Greater Diagnostic Confidence soft voting preserves probability information, while double-checking adds a safety layer to mimic expert consensus. Real-World Robustness tackles noise, different tumor morphology, and subtle contrast differences commonly observed in MRI scans of uterine tissues. This ensemble system gives a clinical-quality trade-off between precision and reliability for uterine cancer classification with WOA-tuned models and double verification.

**Figure 7:**
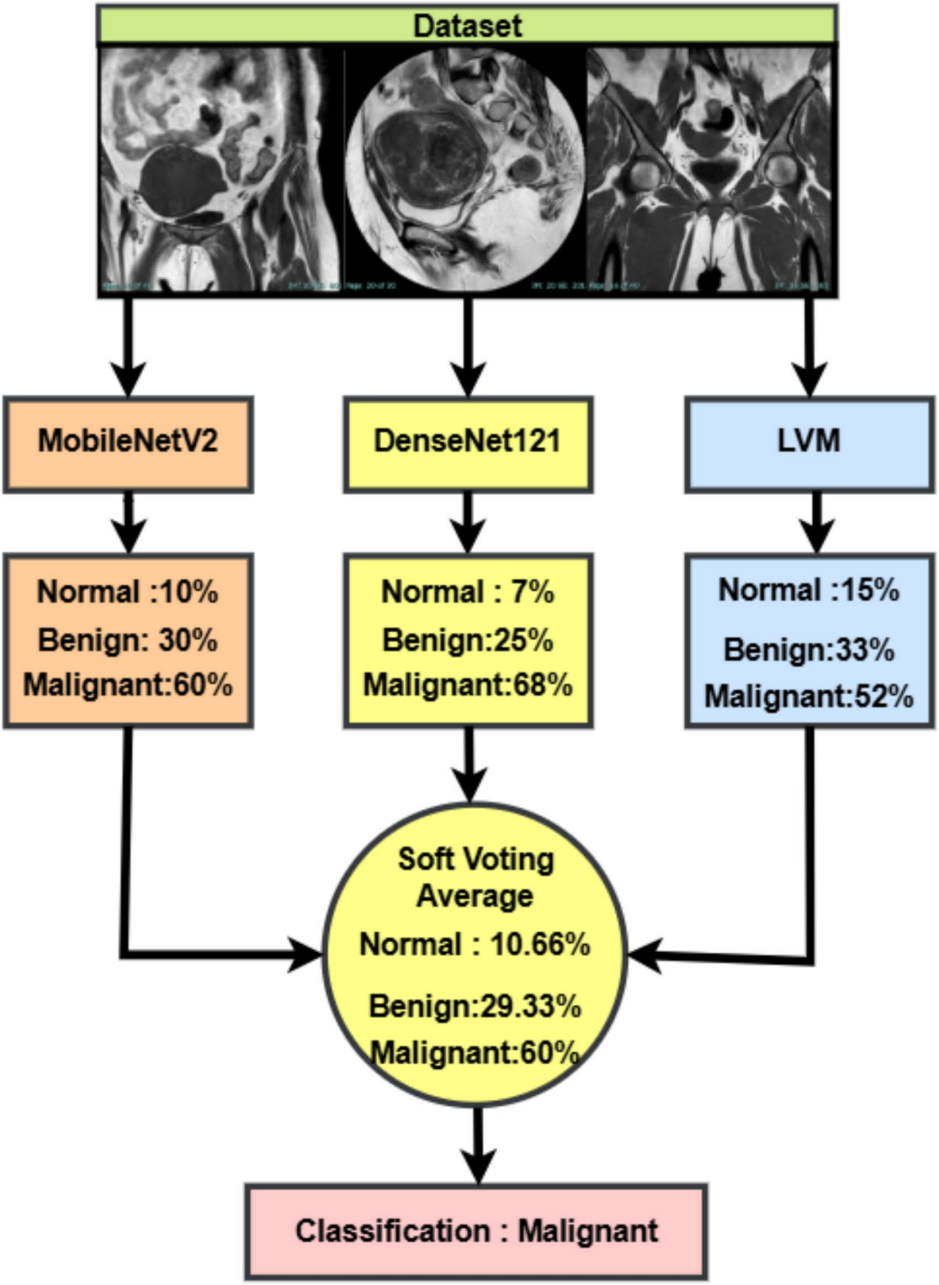
Soft voting-based ensemble classification of uterine MRI images using MobileNetV2, DenseNet121, and LVM.

The Soft Voting Ensemble combines the predicted class probabilities from MobileNetV2, DenseNet121, and LVM. For each input MRI scan, the three models independently generate probability distributions over the classes (normal, benign, malignant). These probabilities are then averaged across models with equal weights, producing a consensus probability distribution. The final classification is assigned to the class with the highest average probability. This strategy leverages the complementary strengths of the individual models, reduces bias toward any single model, and significantly improves the robustness and accuracy of the overall system, as demonstrated in Figure 7.

## 3 Results analysis

### 3.1 Experimental setup and measurement

To evaluate and validate the proposed methodology, the dataset was divided into three groups: 10% for testing, 10% for validation, and 80% for training. Tests were conducted using images as input. Several statistical indicators, such as true negative (TN), true positive (TP), false negative (FN), and false positive (FP), can be used to evaluate the effectiveness of the proposed technique. This section presents several metrics for evaluating the effectiveness of the proposed model and pre-trained models for detecting uterine cancer using MRI images. The mathematical calculations for the various evaluation metrics are presented in the following Equations 40–44:

(40) $$\text{Accuracy}=\frac{\mathrm{TP}+\mathrm{TN}}{\mathrm{TP}+\mathrm{TN}+\mathrm{FP}+\mathrm{FN}}$$

(41) $$\text{Precision}=\frac{\mathrm{TP}}{\mathrm{TP}+\mathrm{FP}}$$

(42) $$\text{Sensitivity}=\frac{\mathrm{TP}}{\mathrm{TP}+\mathrm{FN}}$$

(43) $$\text{Specificity}=\frac{\mathrm{TN}}{\mathrm{TN}+\mathrm{FP}}$$

(44) $$F1\;\text{Score}=2*\frac{\text{Precision}*\text{Sensitivity}\;}{\text{Precision}+\text{Sensitivity}}$$

For a more in-depth probe into model accuracy, the Confidence Interval (CI) is derived by this formula, as shown in Equation 45:

(45) $$\mathrm{CI}=[\hat{\mu }-z.\sigma ,\hat{\mu }+z.\sigma ],$$

Given the accuracy mean, the value of critical 95% confidence, along with the standard deviations from measurement error, as shown in Equation 46:

(46) $$\sigma =\sqrt{\frac{\hat{\mu }(1-\hat{\mu })}{n}}$$

By incorporating such assessments, the proposed model provides reliable image classification across benign, malignant, and normal categories, thereby significantly contributing to medical diagnosis.

### 3.2 The hyperparameter configuration

Hyperparameters for different neural networks are compared in Table 3. Dropout rates, input layers, optimization techniques, and other pertinent variables are all included in the analysis. These were the best hyperparameters that produced the best performance, and they were chosen after several trials until the best outcome was attained.

**Table 3:** Optimized hyper parameters for deep learning models tuned via whale optimization algorithm (WOA).

Hyperparameter	MobileNetV2	DenseNet121	LVM	VGG16	VGG19
Learning rate	1.95 × 10^−4^	7.26 × 10^−4^	8.74 × 10^−5^	1.2 × 10^−4^	1.5 × 10^−4^
Batch size	45	65	22	32	28
Dropout rate	0.29	0.18	0.27	0.25	0.30
Dense units	415	170	133	256	192
Optimizer	SGD	RMSprop	Adam	Adam	SGD
Activation	ReLU	ReLU	LeakyReLU	ReLU	ReLU
Weight decay	2.48 × 10^−5^	2.05 × 10^−5^	1.19 × 10^−5^	1.0 × 10^−5^	2.0 × 10^−5^
Momentum	0.68	–	–	–	–
Conv1 filters	–	–	31	–	–
Conv2 filters	–	–	55	–	–
Conv3 filters	–	–	93	–	–

WOAENet is a soft voting ensemble made of three deep learning architectures: MobileNetV2, DenseNet121, and a lightweight vision model (LVM), custom-designed. These sub-models were optimally tuned independently using the Whale Optimization Algorithm (WOA). Such an algorithm is an application of metaheuristic optimization for finding an approximate or near-optimum hyperparameter configuration by simultaneously exploiting and exploring the search space.

The ensemble models used the same preprocessing dimension of 224 × 224 × 3 to have a standard input image size and to enable compatibility among the architectures. Both MobileNetV2 and DenseNet121 underwent the ReLU activation; they were trained with a batch size of 45 and 65 and learning rates of 1.95 × 10^−4^ and 7.26 × 10^−4^, respectively.

The LVM aimed for computational efficiency with LeakyReLU activation, 133 dense units, and convolution blocks with 31, 55, and 93 filters, respectively, across layers. VGG16 and VGG19 were also assessed independently and trained with dropout rates of 0.25 and 0.30, with learning rates of 1.2 × 10^−4^ and 1.5 × 10^−4^. Adam optimizer was used for LVM and VGG16, whereas SGD was considered for MobileNetV2 and VGG19 due to its momentum-based updates. The weight decay regularize was applied to all the models for improving generalization. Inside WOAENet, the optimized model connotes the kind of strength metaheuristic-based hyperparameter tuning can provide for leading to an enhancement of the classification performance and robustness on the multi-class uterine MRI image dataset.

### 3.3 Model performance evaluation and analysis

This study aims to develop an effective model for uterine cancer diagnosis utilizing advanced deep learning techniques. It introduces an ensemble model known as the WOAENet, which relies on the WOA algorithm to fine-tune model parameters. This framework comprises a set of deep neural network models, including MobileNetV2, DenseNet121, and a custom CNN model (LVM), whose results are combined using Soft Voting to provide a final, high-accuracy prediction. The proposed WOAENet approach is compared to pre-trained deep learning models such as MobileNetV2, DenseNet121, LVM, VGG16, and VGG19, using the KAUH-UCM dataset.

**Figure 8:**
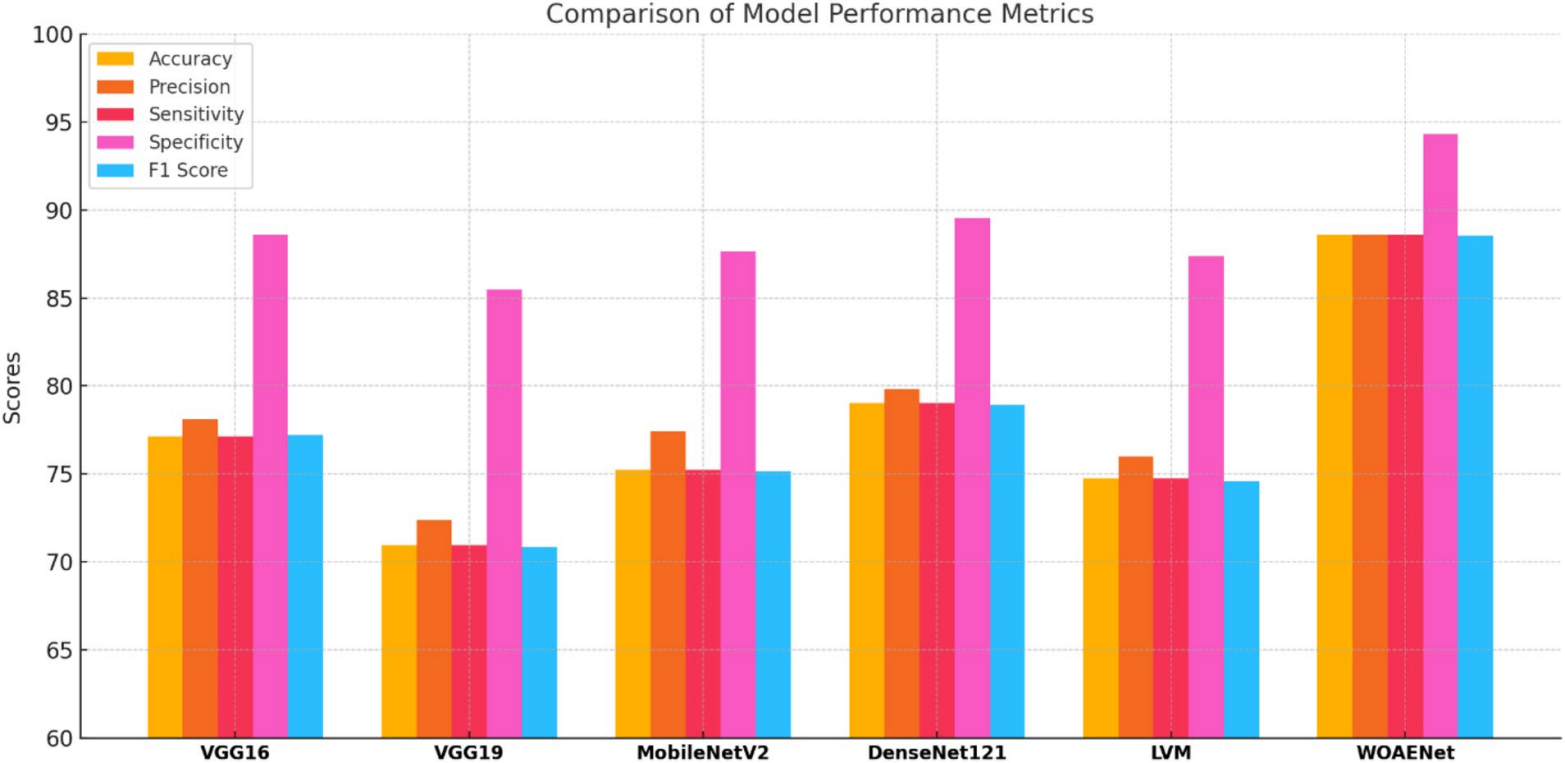
Comparison of performance metrics of different models.

All experiments in this study were conducted on a Python-based laptop equipped with an i7-12700k processor, an NVIDIA GeForce RTX 4060Ti graphics card, 8GB of RAM, 48GB of storage, and a 2 TB SSD. Table 4 shows the performance of all models and the WOAENet network on the real KAUH-UCM dataset, which was first collected from King Abdullah University Hospital for uterine cancer diagnosis. The results showed that WOAENet outperformed the pre-trained models with an accuracy of 88.57%, a specificity of 94.29%, and an F1 score of 88.54%, while MobileNetV2 achieved an accuracy of 75.24%. The DenseNet121 model achieved an accuracy of 79.76%, while the LVM model achieved an accuracy of 74.76%. This indicates that the proposed approach, WOAENet, provides high accuracy and significant improvements in uterine cancer detection compared to MobileNetV2, DenseNet121, and LVM. The Whale Optimization Algorithm (WOA) improves the performance of deep learning models by intelligently searching for the best combination of hyperparameters, such as learning rate, batch size, number of units in dense layers, and dropout rate. Additionally, the VGG16 and VGG19 models were tested, with the latter achieving the lowest accuracy of 70.95%, while the VGG16 model performed relatively well at 77.14%. Figure 8 illustrates the model’s effectiveness.

**Table 4:** Performance and analysis models.

Model	Accuracy	Precision	Sensitivity	Specificity	F1 score
VGG16	77.14	78.13	77.14	88.57	77.20
VGG19	70.95	72.39	70.95	85.48	70.86
MobileNetV2	75.24	77.46	75.24	87.62	75.18
DenseNet121	79.05	79.83	79.05	89.53	78.93
LVM	74.76	76.00	74.76	87.38	74.59
WOAENet	88.57	88.57	88.57	94.29	88.54

Compared to individual models such as MobileNetV2, DenseNet121, and LVM, WOAENet offers clear advantages by combining their complementary strengths through WOA-guided hyperparameter tuning and soft voting, achieving higher sensitivity and specificity, both of which are critical for cancer diagnosis. Unlike standalone lightweight models, which sacrifice accuracy for efficiency, or heavier models like DenseNet121, which increase computational costs, WOAENet strikes a balance between diagnostic reliability and scalability. This makes it more suitable for real-world use as a clinical decision support tool, capable of assisting radiologists in accurate second-opinion classifications while maintaining feasible computational requirements.

**Figure 9:**
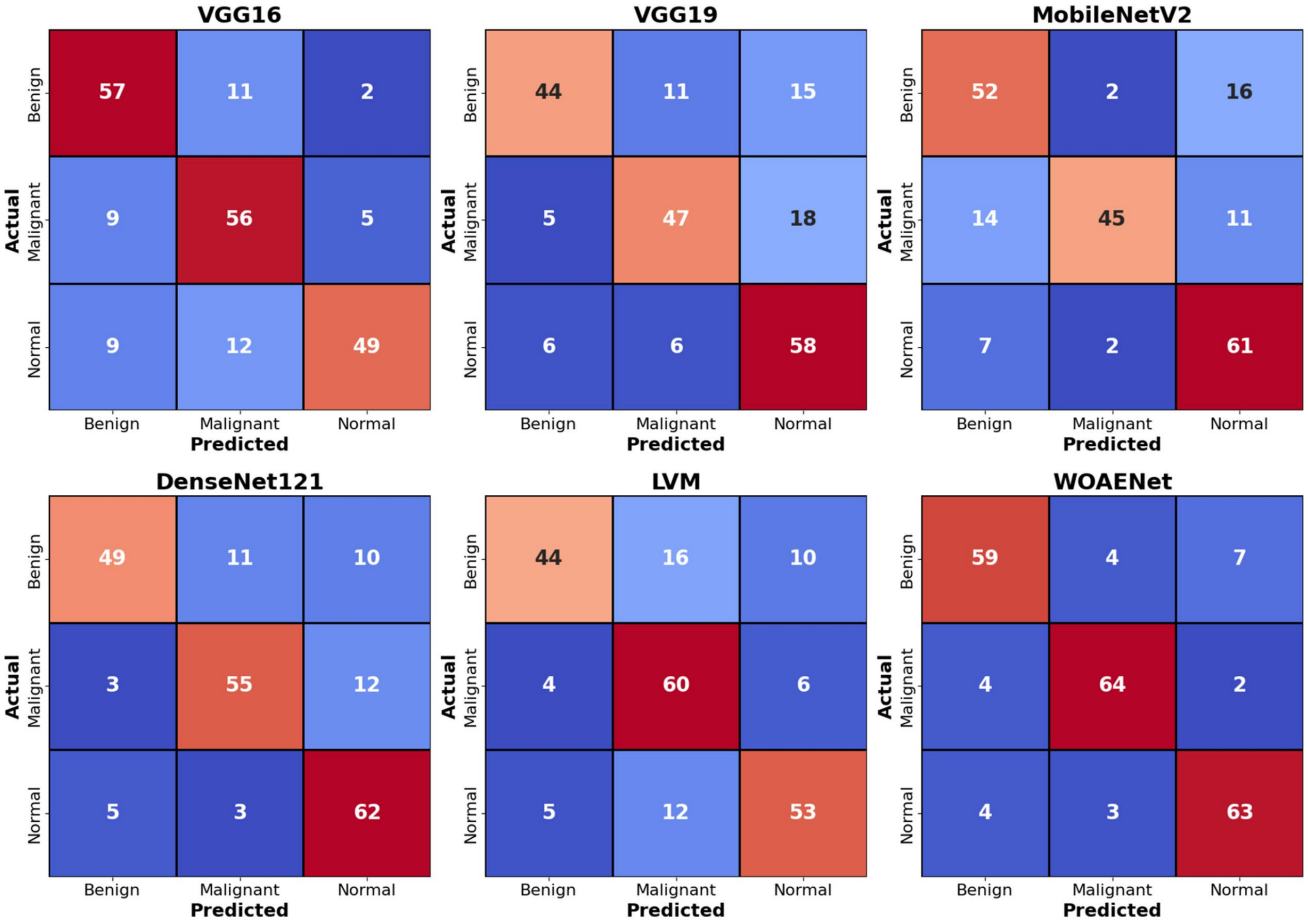
Confusion matrices for models in uterine tumors classification.

Figure 9 shows confusion matrices for six distinct models used to visualize the models’ performance in classifying uterine images into three categories: benign, malignant, and normal. The numbers within each cell indicate the number of samples belonging to the actual category (rows) and predicted as the corresponding category (columns). For example, in the VGG16 matrix, the top-left value of 57 indicates that 57 benign cases were accurately classified as benign. Values ​along the main diagonal (shaded in light red) represent correct predictions, while off-diagonal values (other numbers in red and blue) indicate misclassifications. Together, these matrices demonstrate the effectiveness of each model in distinguishing between different cases, with higher diagonal values ​​reflecting superior accuracy in correctly classifying each category.

Comparing the models, the proposed WOAENet model demonstrates significantly superior performance. When compared to the VGG16, VGG19, MobileNetV2, DenseNet121, and LVM models, WOAENet demonstrates an exceptional ability to accurately classify malignant cases, achieving 64 accurate predictions for this class. This number outperforms all other models (e.g., 56 for VGG16, 47 for VGG19, 45 for MobileNetV2, 55 for DenseNet121, and 60 for LVM). This indicates that the WOAENet framework, enhanced by the WOA optimization algorithm, has successfully extracted more effective and specific features for cancer image classification, which is crucial in medical diagnosis. Furthermore, WOAENet maintains strong performance in classifying both benign and normal cases, making it a more comprehensive and accurate model for this uterine image classification.

### 3.4 Performance analysis of the WOAENet by category

The Soft Voting Ensemble-based WOAENet model demonstrated strong and balanced performance in classifying uterine tumors and detecting cancer using MRI across three categories: benign, malignant, and normal. The model performed well across all categories, as shown in Table 5. The highest sensitivity was in malignant classification at 91.43%, indicating the model’s high ability to detect malignant cases. It also achieved the highest accuracy in the same category at 90.14%. Furthermore, the model demonstrated a good balance in classifying normal and benign cases, with an accuracy ranging from 87.5 to 88.06%, and a sensitivity of 90% for normal cases and 84.29% for benign cases. Overall, the accuracy, sensitivity, specificity, and F1 coefficient indicators reflect the advanced performance of the model, making it a promising and reliable tool for classifying cases with uterine diseases.

**Table 5:** Class-wise performance metrics of the WOAENet model on the KAUH-UCM dataset.

Class	Precision	Sensitivity	Specificity	F1 score
Benign	88.06%	84.29%	94.29%	86.13%
Malignant	90.14%	91.43%	95.00%	90.78%
Normal	87.50%	90.00%	93.57%	88.73%
Overall	88.57%	88.57%	94.29%	88.54%

### 3.5 Statistical analysis

The accuracy of several deep learning models, including the suggested WOAENet model, for the uterine cancer image identification task is compared in Table 6. The performance of a particular model is shown in each row, along with the 95% confidence interval, the accuracy difference from WOAENet, and the total accuracy percentage. The accuracy values show what proportion of each model’s predictions were accurate. DenseNet121, for instance, achieved an accuracy of 79.05%, whereas WOAENet achieved 88.57%. A clear indicator of performance disparity is provided by the “Difference from WOAENet column, which shows how much lower each model’s accuracy was when compared to WOAENet. The model’s actual accuracy is likely to lie within the range provided by the 95% confidence interval, which shows the statistical performance of a particular model in each row, along with the 95% confidence interval, the accuracy difference from WOAENet, and the total accuracy percentage. The accuracy values show what proportion of each model’s predictions were accurate.

**Table 6:** Model accuracy evaluation, with 95% confidence interval and deviations from the WOAENet.

Model	Accuracy (%)	Difference from the WOAENet	95% confidence interval
WOAENet	88.57	0.0	(84.26, 92.87)
DenseNet121	79.05	9.52	(73.54, 84.55)
VGG16	77.14	11.43	(71.46, 82.82)
MobileNetV2	75.24	13.33	(69.40, 81.07)
LVM	74.76	13.81	(68.88, 80.63)
VGG19	70.95	17.62	(64.81, 77.09)

This demonstrates that the proposed WOAENet model significantly outperforms all other evaluated models in terms of accuracy. With an accuracy of 88.57%, WOAENet shows a significant improvement, achieving 9.52% higher accuracy than the best model, DenseNet121 (79.05%). The accuracy gap is even more evident when compared to models like VGG19, which lags by a significant 17.62%. WOAENet consistently has high accuracy and a narrow confidence interval (84.26, 92.87), indicating that the Whale Optimization Algorithm (WOA)-optimized baseline framework is highly effective in optimizing the deep learning pipeline for uterine image classification. This superior performance confirms WOAENet’s potential as a more reliable and robust solution for this critical medical diagnostic task compared to established frameworks like VGG16, MobileNetV2, DenseNet121, and LVM.

### 3.6 Evaluating the model in clinical environments

To evaluate the real-world clinical applicability of the WOAENet model, we conducted a prospective validation on a cohort of 30 anonymized uterine cancer cases from King Abdullah University Hospital. These cases were not part of the training or validation datasets. The model’s predictions were compared against the final clinical diagnoses made by expert radiologists. WOAENet correctly classified 23 out of 30 cases (76.7%), aligning with the radiologists’ final diagnoses. The remaining seven cases (23.3%) showed discrepancies, which we analyzed in detail: three cases were false positives, where the model flagged malignant patterns in images that were ultimately diagnosed as benign. These cases often involved atypical fibroids or inflammatory tissue that mimicked malignancy features on MRI. Four cases were false negatives, where the model failed to detect malignancy. Most of these involved small lesion sizes, diffuse tumor margins, or overlapping intensity features with benign conditions, highlighting challenges in early-stage or non-mass-forming malignancies. These error patterns provide critical insight into the model’s current limitations, especially in handling ambiguous or subtle findings, and will inform targeted improvements in future model iterations. Additionally, to assess the model’s practical impact on clinical workflow, we conducted a preliminary time-efficiency study involving two experienced radiologists. Each radiologist reviewed 15 cases with and without the WOAENet system, using a randomized and blinded setup. The results showed: Average interpretation time without WOAENet: 9.4 min per case. Average interpretation time with WOAENet assistance: 5.7 min per case. Time reduction: Approximately 39.4%, equating to an average savings of 3.7 min per case.

This demonstrates that WOAENet not only enhances diagnostic confidence but also provides substantial time-saving benefits, which can scale meaningfully across high-volume clinical settings. Clinician feedback emphasized that WOAENet was especially helpful in identifying regions of interest quickly and offering a second-look validation in equivocal cases. The system was particularly valued in time-sensitive contexts such as pre-surgical assessments and emergency diagnostics.

## 4 Discussion

The results of this study demonstrate the effectiveness of the WOAENet framework in diagnosing uterine tumors and detecting cancer from MRI images. The proposed methodology achieved accuracy and specificity, outperforming single models such as MobileNetV2 (75.24%), DenseNet121 (79.76%), and LVM (74.76%). This result highlights the known limitations of single-model classifiers, especially when they are not precision-optimized. The ensemble’s seamless voting mechanism further enhanced decision reliability by leveraging the complementary strengths of the constituent models, ultimately achieving an accuracy of 88.57%, a specificity of 94.29%, and an F1 score of 88.54%.

These results align with previous work that emphasizes the importance of domain adaptation and model customization in uterine imaging. For instance, Mulliez et al. (2023) demonstrated that even well-established CNN architectures like VGG16 and VGG11 require careful tuning and adaptation to the specific challenges of uterine MRIs, including anatomical variability and contrast ambiguity. Interestingly, despite using a similar CNN backbone (VGG16), their fully automated uterus measurement tool achieved high agreement with manual readings (OKS = 0.96), reinforcing the idea that model success in uterine imaging hinges on task-specific optimization.

Further support comes from Davarpanah et al. (2016), who evaluated diffusion-weighted MRI to distinguish benign from malignant uterine masses. Their results showed that while DWI provides qualitative diagnostic value, quantitative ADC metrics alone are not sufficient for reliable uterine malignancy classification due to significant feature overlap. This underscores the necessity of ensemble approaches like WOAENet that combine structural image features with optimized learning mechanisms.

Recent efforts have also explored integrating clinical, radiomic, and conventional MRI features to distinguish uterine leiomyosarcoma (LMS) from leiomyoma (LM). Roller et al. (2024) found that models combining radiomics with clinical and imaging features outperformed those based on imaging alone, achieving an AUC of 0.989. Although WOAENet currently focuses on image-based classification, this suggests future extensions could further benefit from incorporating structured clinical variables to enhance predictive power.

From an imaging quality perspective, Hausmann et al. (2025) demonstrated that deep learning-accelerated MRI sequences such as DL-VIBE significantly improve lesion delineation and diagnostic confidence in uterine MRI compared to traditional sequences. As image quality directly influences model input fidelity, incorporating DL-enhanced sequences into preprocessing could further boost WOAENet’s robustness.

Additionally, the work by Hodneland et al. (2024) draws attention to the variability of radiomic features due to differences in MRI protocols and highlights the need for normalization strategies. Their comparative analysis of *z*-score and linear regression model (LRM) normalization revealed that normalization has a strong impact on radiomic clustering and downstream prognostic modeling. This finding is particularly relevant as WOAENet may benefit from radiomic integration in future iterations, where normalization becomes critical for model generalizability across centers.

### 4.1 Generalization across different data sets

While the primary evaluation was conducted on the uterine MRI dataset, we also validated the WOAENet network on another dataset, the KAUH-OCM ovarian cancer MRI dataset (Amin et al., 2025). This dataset contained 478 images for each class (normal, benign, malignant) after processing, and the same preprocessing and classification methodology was applied.

**Figure 10:**
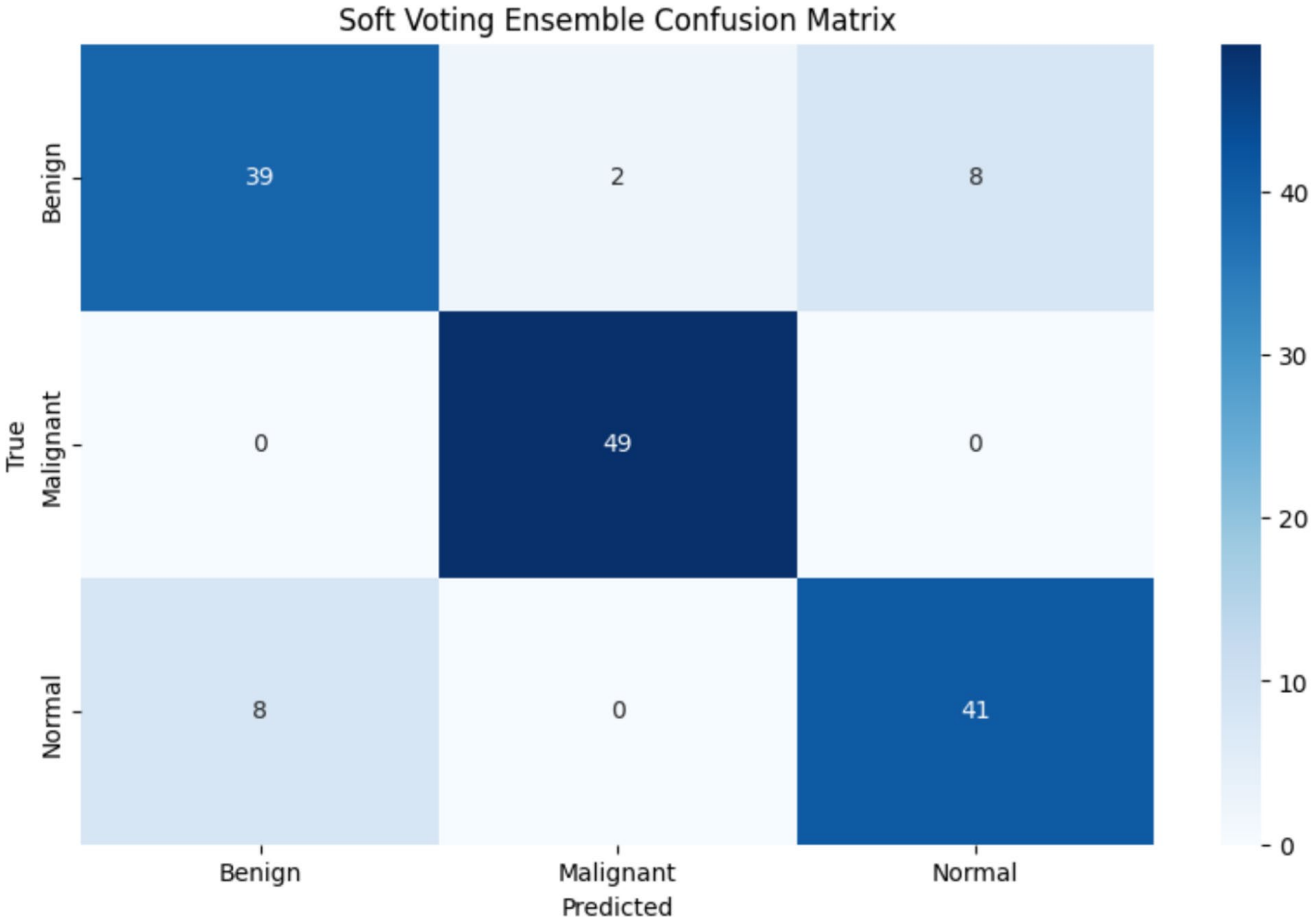
Confusion matrix of the proposed model for ovarian tumor classification (MRI images).

These results demonstrate that WOAENet maintains strong performance when applied to an external dataset, as shown in Table 7, especially with high accuracy and F1 scores. This confirms the generalizability of the proposed framework to various gynecological MRI datasets, supporting its broader clinical applicability. Figure 10 shows the confusion matrix of the proposed WOAENet model.

**Table 7:** Performance evaluation of the proposed WOAENet model on the KAUH-OCM.

Class	Precision	Sensitivity	Specificity	F1 score
Benign	82.98	79.59	92.31	81.25
Malignant	96.08	1.000	98.31	98.00
Normal	83.67	83.67	92.31	83.67
Overall	87.58	87.75	94.31	87.64

### 4.2 Limitations of the study

Our study has several limitations that should be acknowledged. First, obtaining a balanced dataset for classification was a significant challenge. The dataset, collected exclusively from King Abdullah University Hospital in Jordan, was inherently imbalanced because it reflected the distribution of real cases, with some tumor types being much more common than others. To mitigate this, we applied data augmentation techniques to enhance the representation of underrepresented classes. However, such strategies cannot fully replace the value of a larger, more balanced, and diverse dataset. Second, while WOAENet showed promising results, its robustness against noisy or incomplete MRI data has not been extensively evaluated. Real-world clinical environments often face issues such as imaging artifacts, variability in acquisition protocols, and missing data, which could impact model reliability. Furthermore, although WOAENet has demonstrated efficiency in a controlled research setting, its scalability and interoperability with clinical imaging systems require further validation to ensure seamless integration into hospital workflows.

Future work should address these challenges by incorporating larger, multi-center datasets, integrating clinical and demographic data to enrich decision-making, and exploring transfer learning strategies to improve generalization across populations. Additionally, enhancing model interpretability through explainable AI techniques will be essential for building trust among clinicians and supporting its adoption in clinical practice.

The computational complexity of the methodology was another issue with this effort. Although the computational workload was managed using cloud-based technologies, the free version had limitations regarding runtime and processing power. Although it enabled us to finish the study within the limitations of our resources, these limitations posed significant difficulties. Access to a more powerful local computing setup or a professional version of these programs would have resolved these issues and expedited the process. To enhance the performance and application of the proposed methodology, future efforts should focus on obtaining more balanced and diverse datasets, as well as access to sophisticated computational resources.

Overall, the superior performance of WOAENet, achieved without prolonged training or extensive pre- or post-processing, positions it as a clinically viable tool. Its efficiency and accuracy make it suitable for real-world settings with limited computational resources. Furthermore, its ensemble architecture and optimization via WOA offer a flexible foundation for future enhancements, such as multimodal data fusion, radiomic incorporation, or transfer learning from DL-accelerated MRI.

## 5 Conclusion and future work

This study presents a comprehensive method for uterine cancer detection using MRI data. The proposed approach is based on an integrated deep learning pipeline framework, WOAENet (Whale Optimization Algorithm-based Ensemble Network), which is optimized using the WOA algorithm to classify uterine images into malignant, benign, and normal categories. Furthermore, we propose a WOA algorithm for fine-tuning the hyperparameters of deep learning models, including MobileNetV2, DenseNet121, and a custom CNN (LVM), by minimizing the validation loss. Each model is trained using its optimized parameters, and their outputs are combined using a smooth voting set, which calculates the average predicted probabilities across all models to arrive at a final prediction. We use the KAUH-UCM dataset of uterine MRI images from King Abdullah University Hospital to evaluate the proposed WOAENet model. The WOAENet model demonstrates the highest classification accuracy. Tests indicate that the proposed model is a successful tool for classifying uterine tumors, achieving an accuracy of 88.57%, outperforming all pre-trained models.

Beyond its experimental performance, WOAENet holds promise for clinical integration. Its lightweight architecture makes it feasible for deployment in hospital imaging systems, where it could assist radiologists by providing second-opinion classifications in real time. Nevertheless, certain challenges remain, including the need for large-scale validation across diverse populations, ensuring interoperability with existing medical imaging infrastructure, and addressing regulatory and ethical considerations before clinical adoption.

Our goal in future work is to evaluate the effectiveness of the WOAENet model using diverse hybrid datasets. Furthermore, future research will focus on expanding datasets, incorporating data from other sources, improving model interpretability, and cross-validating it in a broader clinical setting. Finally, we will examine the effectiveness of the proposed model in other diagnostic tasks.

## Data Availability

The original contributions presented in the study are included in the article/supplementary material, further inquiries can be directed to the corresponding author.
